# Stability, Boundedness, and Lagrange Stability of Fractional Differential Equations with Initial Time Difference

**DOI:** 10.1155/2014/939027

**Published:** 2014-02-12

**Authors:** Muhammed Çiçek, Coşkun Yakar, Bülent Oğur

**Affiliations:** Department of Mathematics, Gebze Institute of Technology, Gebze, Kocaeli 141-41400, Turkey

## Abstract

Differential inequalities, comparison results, and sufficient conditions on initial time difference stability, boundedness, and Lagrange stability for fractional differential systems have been evaluated.

## 1. Introduction

The problem of stability of solutions is one of the major problems in the theory of differential equations. Lyapunov function and the Lyapunov direct method allow us to obtain sufficient conditions for the stability of a system without explicitly solving the differential equations [1, 2]. The method generalizes the idea which shows that the system is stable if there are some Lyapunov function candidates for the system.

Only a few decades ago, it was realized that fractional calculus provides an attractive tool for modelling the real world problems. The differentiation and integration of arbitrary orders have found applications in diverse fields of science and engineering like viscoelasticity, electrochemistry, diffusion processes, control theory, heat conduction, electricity, mechanics, chaos, and fractals [3–5]. Recently, some attention has been drawn on stability analysis of fractional differential equations (FDE) [6–9].

In practical situations, it is possible to have not only a change in initial position but also in initial time because of all kinds of disturbed factors. When we do consider such a deviation in initial time, it causes measuring the difference between any two different solutions starting with different initial times. From this point of view, several studies have been made on this problem to explore the stability and boundedness, criteria for differential systems relative to initial time difference (ITD) by using variation of parameters and differential inequalities technique [10–13]. In this paper, the stability and boundedness criteria for FDE relative to initial time difference have been investigated by using comparison method. In Section 2 the differences between classical notion of stability and the notion of initial time difference (ITD) stability have been discussed and compared by giving basic definitions. In Section 3 new differential inequalities and comparison results relative to initial time difference are obtained. Then, we investigate ITD stability, boundedness and Lagrange stability by using results obtained in Section 3. Lastly the conclusions are given in Section 4.

## 2. Fractional Calculus

Fractional calculus generalizes the derivative and the integral of a function to a noninteger order [3, 4, 14]. Although there are several definitions of fractional derivatives and fractional integrals, only the relative ones are given below. Let *f* : [*a*, *b*] → ℝ be a function.

**Definition 1**

The fractional integral (or the Riemann-Liouville (RL) type integral) of order *q* > 0 is defined as

(1) $$\begin{array}{c} I_{a}^{q}f\left(t\right)=\frac{1}{\Gamma \left(q\right)}\int_{a}^{t}{\left(t-s\right)}^{q-1}f\left(s\right)ds. \end{array}$$

**Definition 2**

The RL fractional derivatives of order *q* ∈ [*n* − 1, *n*) of *f*(*t*) are defined as

(2) Dtqaf(t)=dndtnItn−qaf(t)=1Γ(n−q)dndtn∫at(t−s)n−q−1f(s)ds.

**Definition 3**

The Caputo fractional derivative of order *q* ∈ (*n* − 1, *n*] of *f*(*t*) is defined as

(3) Dtqacf(t)=Ian−q[dndtnf(t)]=1Γ(n−q)∫at(t−s)n−q−1f(n)(s)ds.

To simplify the notations we will use *I* ^*q*^, *D* ^*q*^, and ^*c*^ *D* ^*q*^ instead of *I*_*a*_^*q*^, _*a*_ *D*_*t*_^*q*^, and _*a*_^*c*^ *D*_*t*_^*q*^, respectively. There are some relation between these fractional integral and derivatives. For details please see [3, 4, 14].

**Property 1**

- *I* ^*α*^(*I* ^*β*^ *f*(*t*)) = *I* ^*α*+*β*^ *f*(*t*).
- *ℒ*[*D* ^*q*^ *f*(*t*)] = *s* ^*q*^ *X*(*s*) − ∑_*k*=0_^*n*−1^ *s* ^*k*^[*D* ^*q*−*k*−1^ *f*(*t*)]_*t*=0_, where *D* ^*q*^ represents _0_ *D*_*t*_^*q*^.
- *ℒ*[^*c*^ *D* ^*q*^ *f*(*t*)] = *s* ^*q*^ *X*(*s*) − ∑_*k*=0_^*n*−1^ *s* ^*q*−*k*−1^ *f* ^(*k*)^(0), where ^*c*^ *D* ^*q*^ represents _0_^*c*^ *D*_*t*_^*q*^.
- ^*c*^ *D* ^*q*^ *I* ^*q*^ *f*(*t*) = *f*(*t*) and *D* ^*q*^ *I* ^*q*^ *f*(*t*) = *f*(*t*).
- *I* ^*q*^ *D* ^*q*^ *f*(*t*) = *f*(*t*) − ∑_*k*=1_^*n*−1^[*D* ^*q*−*k*^ *f*(*t*)]_*t*=*a*_((*t* − *a*)^*q*−*k*^/Γ(*k* − *q* + 1)) and *I* ^*q*^ ^*c*^ *D* ^*q*^ *f*(*t*) = *f*(*t*)‒∑_*k*=0_^*n*−1^((*t* − *a*)^*k*^/*k*!)*f* ^(*k*)^(*a*).
- ^*c*^ *D* ^*q*^ *f*(*t*) = *D* ^*q*^(*f*(*t*) − ∑_*k*=0_^*n*−1^((*t* − *a*)^*k*^/*k*!)*f* ^(*k*)^(*a*)).
- *D* ^*q*^ *C* = *C*(*t* − *a*)^−*q*^/Γ(1 − *q*) and ^*c*^ *D* ^*q*^ *C* = 0, where *C* is arbitrary constant.

After giving definition and properties of fractional integral and derivatives, we consider fractional order IVP with (RL) and Caputo derivative, respectively;

(4) Dqx(t)=f(t,x),x(t0)=x0=Γ(q)x(t)(t−t0)1−q|t=t0,Dqcx(t)=f(t,x)x(t0)=x0,

where 0 < *q* < 1, *f* ∈ *C*[*J* × ℝ, ℝ] and *J* = [*t*_0_, *T*]. Then IVP's are equivalent to the following Volterra fractional integral equations:

(5) $$\begin{array}{c} x\left(t\right)=\frac{x^{0}{\left(t-t_{0}\right)}^{q-1}}{\Gamma \left(q\right)}+\frac{1}{\Gamma \left(q\right)}\int_{t_{0}}^{t}{\left(t-s\right)}^{q-1}f\left(s,x\left(s\right)\right)ds, \end{array}$$

(6) $$\begin{array}{c} x\left(t\right)=x_{0}+{\frac{1}{\Gamma \left(q\right)}}_{t}\int_{t_{0}}^{t}{\left(t-s\right)}^{q-1}f\left(s,x\left(s\right)\right)ds, \end{array}$$

respectively. In [15] authors develop a relation between the solutions of Caputo fractional differential equations and those of (RL) fractional differential equations.

## 3. Stability versus Initial Time Difference Stability

Consider the IVP for the system of nonlinear fractional differential equation

(7) Dqcx(t)=f(t,x),x(t0)=x0,

where *t*_0_ ∈ ℝ_+_, *f* ∈ *C*[ℝ_+_ × ℝ^*n*^, ℝ^*n*^] and 0 < *q* < 1. Suppose that the function *f* is smooth enough to guarantee existence, uniqueness, and continuous dependence of solutions of IVP (7). Denote by *x*(*t*) = *x*(*t*, *t*_0_, *x*_0_) the solution of (7). Consider also the initial value problem at a different initial data; that is, let *τ*_0_ ∈ ℝ_+_ and *y*(*t*) = *y*(*t*, *τ*_0_, *y*_0_) be the solution of the system (7). Assume that *x*(*t*) = *x*(*t*, *t*_0_, *x*_0_) is the solution on which we shall study stability and boundedness criteria with respect to it. Set *η* = *τ*_0_ − *t*_0_ > 0 and denote *S*(*ρ*) = {*x* ∈ ℝ^*n*^ : ||*x*|| < *ρ*}.

Before giving our comparison theorem, stability, boundedness criteria, and Lagrange stability for FDE we need to introduce the following definitions.

**Definition 4**

The solution *x*(*t*) = *x*(*t*, *t*_0_, *x*_0_) of (7) is said to be

(S1) stable with ITD, if given *ϵ* > 0 and *t*_0_ ∈ ℝ_+_; there exist *δ* = *δ*(*ϵ*, *t*_0_) > 0 and $\tilde{\delta }=\tilde{\delta }(\epsilon ,t_{0})>0$ such that
  (8) ||y0−x0||<δ,|η|<δ~  imply ||y(t+η,τ0,y0)−x(t,t0,x0)||<ϵfor  t≥t0;
(S2) uniformly stable with ITD, if (*S*_1_) holds with *δ* and $\tilde{\delta }$ independent of *t*_0_.

**Definition 5**

The system (7) is said to be

(B1) equibounded with ITD, if given *α* > 0 and *t*_0_ ∈ ℝ_+_; there exist $\tilde{\delta }=\tilde{\delta }(\alpha ,t_{0})>0$ and *β* = *β*(*α*, *t*_0_) > 0 such that
  (9) ||y0−x0||≤α,|η|<δ~  imply ||y(t+η,τ0,y0)−x(t,t0,x0)||<β t≥t0;
(B2) uniformly bounded with ITD, if (*B*_1_) holds with $\tilde{\delta }$ and *β* independent of *t*_0_;
(A1) attractive in the large with ITD, if for every *ϵ* > 0 and each *α* > 0 there exists $\tilde{\delta }=\tilde{\delta }(\epsilon ,\alpha ,t_{0})$ and *T* = *T*(*ϵ*, *α*, *t*_0_) such that
  (10) ||y0−x0||≤α,|η|<δ~  imply ||y(t+η,τ0,y0)−x(t,t0,x0)||<ϵfor  t≥t0+T;
(L1) Lagrange stable if (B1) and (A1) hold.

**Definition 6**

A function *a* is said to belong to the class *𝒦* such that

(11) $$\begin{array}{c} 𝒦=\left[a\in C\left[{\mathbb{R}}_{+},{\mathbb{R}}_{+}\right]:a\left(0\right)=0\;\text{and}\;a\;\text{is}\;\text{strictly}\;\text{increasing}\right]. \end{array}$$

**Definition 7**

We define the generalized derivative with respect to the system (7) as follows:

(12) D+qcV(t,y~−x) =lim⁡h→0+⁡sup⁡1hq[V(t,y~−x) −V(t−h,y~−x−hq ×(f(t+η,y~)−f(t,x)))]

for $(t,\tilde{y}-x)\in {\mathbb{R}}_{+}\times {\mathbb{R}}^{n}$, where *x* = *x*(*t*) = *x*(*t*, *t*_0_, *x*_0_) is the solution of (7) and $\tilde{y}=\tilde{y}(t,t_{0},y_{0})=y(t+\eta ,\tau_{0},y_{0})$, where *y*(*t*, *τ*_0_, *y*_0_) is the solution of (7) and *η* = *τ*_0_ − *t*_0_ > 0.

The stability with ITD gives us an opportunity to compare solutions of FDE where both initial time and position are different. In the case of differential equation, stability with ITD is studied in [10–13, 16]. We will give a brief overview of both concepts of stability.

**Case 1 (classical notion of stability)**

Let *x*(*t*) = *x*(*t*, *t*_0_, *x*_0_) be a solution of (7). Study the stability of *x*(*t*). Consider the solution *Y*(*t*) = *Y*(*t*, *t*_0_, *y*_0_) of (7). Set the IVP as

(13) Dqcz=f~(t,z),z(t0)=y0−x0,

where $\tilde{f}(t,z)=f(t,z+x(t))-f(t,x(t))$. The function *z*(*t*; *t*_0_, *y*_0_, *x*_0_) = *Y*(*t*) − *x*(*t*) is a solution of the IVP (13).

The IVP (13) has a zero solution and the study of stability properties of the nonzero solution *x*(*t*) of (7) is reduced to the stability of the zero solution of transformed system (13).

**Case 2 (stability with ITD)**

Study the stability with initial time difference of *x*(*t*). Consider the solution of (7) with different initial data as *y*(*t*) = *y*(*t*, *τ*_0_, *y*_0_). Set the IVP as

(14) Dqcz=f~(t,z),z(t0)=y0−x0,

where $\tilde{f}(t,z;\eta )=f(t+\eta ,z+x(t))-f(t,x(t))$. Then *z*(*t*; *t*_0_, *τ*_0_, *y*_0_, *x*_0_) = *y*(*t* + *η*) − *x*(*t*) is a solution of (14). The system (14) has no zero solution since $\tilde{f}(t,0)=f(t+\eta ,x(t))-f(t,x(t))\neq 0$. Therefore, in this case the study of stability with ITD of *x*(*t*) could not be reduced to the study of stability of the zero solution of an appropriate fractional differential system.

## 4. Main Results

### 4.1. Differential Inequalities and Comparison Results

#### 4.1.1. Differential Inequalities

**Definition 8 (see [15])**

*m* is said to be *C* ^*q*^ continuous; that is, *m* ∈ *C* ^*q*^([*t*_0_, *T*], ℝ), if and only if the Caputo derivative of ^*c*^ *D* ^*q*^ *m*(*t*) exists and satisfies

(15) Dqcm(t)=1Γ(1−q)∫t0t(t−s)−qm′(s)ds.

**Lemma 9**

Let *m* ∈ *C* ^*q*^([*t*_0_, *T*], ℝ). Suppose that for any *t*_1_ ∈ (*t*_0_, *T*], one has *m*(*t*_1_) = 0 and *m*(*t*) < 0 for *t*_0_ ≤ *t* < *t*_1_; then it follows that

(16) Dqcm(t1)>0.

**Proof**

From the relation between Riemann-Liouville and Caputo fractional derivatives we write

(17) Dqcm(t)=Dq[m(t)−m(t0)]=Dqm(t)−m(t0)(t−t0)−qΓ(1−q).

Since *m*(*t*_0_) < 0 we have *D* ^*q*^ *m*(*t*_0_) < 0. Therefore, we obtain

(18) Dqcm(t)>Dqm(t).

Finally using the lemma for R-L derivative from [6] and (18) implies that

(19) Dqcm(t1)>Dqm(t1)≥0.

**Theorem 10**

Let *v*, *w* ∈ *C* ^*q*^([*t*_0_, *T*], ℝ), *f* ∈ *C*([*t*_0_, *T*] × ℝ, ℝ) and

(20) (i) Dqcv(t)≤f(t,v(t)),(ii) Dqcw(t)>f(t,w(t)), t0≤t≤T.

Then *v*(*t*_0_) < *w*(*t*_0_) implies

(21) $$\begin{array}{c} v\left(t\right)<w\left(t\right),\;t_{0}\leq t\leq T. \end{array}$$

**Proof**

Suppose that relation (21) is false. Then since *v*(*t*_0_) < *w*(*t*_0_) and *v*(*t*), *w*(*t*) are continuous, there exists a *t*_1_ ∈ (*t*_0_, *T*] with *v*(*t*_1_) = *w*(*t*_1_) and *v*(*t*) < *w*(*t*) for *t*_0_ ≤ *t* < *t*_1_. Set *m*(*t*) = *v*(*t*) − *w*(*t*). Then *m*(*t*_1_) = 0 and *m*(*t*) < 0 for *t*_1_ ∈ [*t*_0_, *t*_1_). Hence, the hypothesis of Lemma 9 holds and we conclude that ^*c*^ *D* ^*q*^ *m*(*t*_1_) > 0, which means that ^*c*^ *D* ^*q*^ *v*(*t*_1_) > ^*c*^ *D* ^*q*^ *w*(*t*_1_). Consider

(22) f(t1,v(t1))≥Dqcv(t1)>Dqcw(t1)>f(t1,w(t1))

which is a contradiction. Thus the conclusion of the theorem holds and the proof is complete.

**Theorem 11**

Let *v*, *w* ∈ *C* ^*q*^([*t*_0_, *T*], ℝ), *f* ∈ *C*([*t*_0_, *T*] × ℝ, ℝ) and

(23) (i) Dqcv(t)≤f(t,v(t)),(ii) Dqcw(t)≥f(t,w(t)), t0≤t≤T.

Assume *f* satisfies the Lipschitz condition

(24) $$\begin{array}{c} f\left(t,x\right)-f\left(t,y\right)\leq L\left(x-y\right),\;x\geq y,\;L>0. \end{array}$$

Then, *v*(*t*_0_) ≤ *w*(*t*_0_) implies

(25) $$\begin{array}{c} v\left(t\right)\leq w\left(t\right),\;t_{0}\leq t\leq T. \end{array}$$

**Proof**

We set *w*_*ϵ*_(*t*) = *w*(*t*) + *ϵλ*(*t*), where *λ*(*t*) = *λ*(*t*_0_)*E*_*q*_(2*L*(*t* − *t*_0_)^*q*^) is the solution of linear fractional differential equation ^*c*^ *D* ^*q*^ *λ*(*t*) = 2*Lλ*(*t*), *λ*(*t*_0_) = *λ*_0_. Then from *w*_*ϵ*_(*t*_0_) = *w*(*t*_0_) + *ϵλ*(*t*_0_), we get *w*_*ϵ*_(*t*_0_) > *w*(*t*_0_) ≥ *v*(*t*_0_). In order to get (25), we need to apply Theorem 10 to *v*(*t*) and *w*_*ϵ*_(*t*). Using (*ii*) and Lipschitz condition we have

(26) Dqcwϵ(t)=Dqcw(t)+Dqcλ(t)≥f(t,w(t))−f(t,wϵ(t))+f(t,wϵ(t)) +ϵ2Lλ(t),Dqcwϵ(t)>f(t,wϵ(t)).

Applying now Theorem 10 to *v*(*t*) and *w*_*ϵ*_(*t*), we get *v*(*t*) < *w*_*ϵ*_(*t*) for every *ϵ* > 0 and consequently making *ϵ* → 0, we get the desired estimate (25).

#### 4.1.2. Comparison Results

The most commonly used technique in the theory of differential equations is related to the estimation of a function satisfying a differential inequality by the extremal solutions of the related differential equation. The following theorems give such estimate with initial time difference.

**Theorem 12**

Assume that *m* ∈ *C* ^*q*^([*t*_0_, *T*], ℝ) and

(27) Dqcm(t)≤g(t,m(t)), t0≤t≤T,

where *g* ∈ *C*([*t*_0_, *T*] × ℝ, ℝ). Let *r*(*t*) be the maximal solution of the IVP

(28) Dqcu=g(t,u),  u(t0)=u0,

existing on [*t*_0_, *T*] such that *m*(*t*_0_) ≤ *u*_0_. Then one has

(29) $$\begin{array}{c} m\left(t\right)\leq r\left(t\right),\;t_{0}\leq t\leq T. \end{array}$$

**Proof**

In view of the definition of the maximal solution *r*(*t*), it is enough to prove that *m*(*t*) < *u*(*t*, *ϵ*), *t*_0_ ≤ *t* ≤ *T*, where *u*(*t*, *ϵ*) is any solution of the IVP

(30) Dqcu=g(t,u)+ϵ, with the initial value u(t0)+ϵ,  ϵ>0.

Using Lemma 9, we get *m*(*t*) < *u*(*t*, *ϵ*). And from lim⁡_*ϵ*→0_⁡*u*(*t*, *ϵ*) = *r*(*t*) uniformly on each compact set *t*_0_ ≤ *t* ≤ *T*_0_ < *T*, we get the desired estimate (29).

**Theorem 13**

Assume that

(i) *m* ∈ *C* ^*q*^([*t*_0_, *T*], ℝ_+_), *g* ∈ *C*([*t*_0_, *T*] × ℝ_+_, ℝ) and
  (31) Dqcm(t)≤g(t,m(t)), m(t0)≤w0;
(ii) the maximal solution *r*(*t*) = *r*(*t*, *τ*_0_, *w*_0_) of the IVP
  (32) Dqcw=g(t,w), w(τ0)=w0≥0
  exists for *t* ≥ *τ*_0_;
(iii) *g*(*t*, *w*) is nondecreasing in *t* for each *w* and *τ*_0_ > *t*_0_.

Then (a) *m*(*t*) ≤ *r*(*t* + *η*), *t* ≥ *t*_0_ and (b) *m*(*t* − *η*) ≤ *r*(*t*), *t* ≥ *τ*_0_.

**Proof**

(a) It is well known that if *w*(*t*, *ϵ*) is any solution of

(33) Dqcw=g(t,w)+ϵ, w(τ0)=w0+ϵ for  ϵ>0

sufficiently small then lim⁡_*ϵ*→0_⁡*w*(*t*, *ϵ*) = *r*(*t*, *τ*_0_, *w*_0_) on every compact set [*τ*_0_, *τ*_0_ + *T*].

Setting *w*_0_(*t*, *ϵ*) = *w*(*t* + *η*, *ϵ*), we have *w*_0_(*t*_0_, *ϵ*) = *w*(*t*_0_ + *η*, *ϵ*) = *w*(*τ*_0_, *ϵ*) = *w*_0_ + *ϵ* > *w*_0_ ≥ *m*(*t*_0_). Thus we get *m*(*t*_0_) < *w*_0_(*t*_0_, *ϵ*). On the other hand, using (iii)

(34) Dqcw0(t,ϵ)=g(t+η,w0(t,ϵ))+ϵ>g(t+η,w0(t,ϵ))>g(t,w0(t,ϵ)), t≥t0.

Then we get *m*(*t*) < *w*_0_(*t*, *ϵ*),  *t* ≥ *t*_0_ and hence it follows that *m*(*t*) ≤ *r*(*t* + *η*),  *t* ≥ *t*_0_.

(b) We set *m*_0_(*t*) = *m*(*t* − *η*) so that *m*_0_(*τ*_0_) = *m*(*t*_0_) ≤ *w*_0_ < *w*_0_ + *ϵ* and

(35) Dqcm0(t)≤g(t−η,m0(t))<g(t,m0(t)), t≥τ0.

Then we get *m*_0_(*t*) < *w*(*t*, *ϵ*), *t* ≥ *τ*_0_. The conclusion follows taking the limit as *ϵ* → 0. The proof of theorem is complete.

In the following theorem, we obtain a comparison result in terms of Lyapunov-like functions with ITD.

**Theorem 14**

Assume that

(i) *V* ∈ *C*[ℝ_+_ × ℝ^*n*^, ℝ_+_], *V*(*t*, *x*) is locally Lipschitzian in *x* ∈ ℝ^*n*^, *g* ∈ *C*[ℝ_+_^2^, ℝ] and
  (36) D+qcV(t,y(t+η)−x(t))≤g(t,V(t,y(t+η)−x(t)));
(ii) the maximal solution *r*(*t*) = *r*(*t*, *τ*_0_, *u*_0_) of the fractional scalar differential equation
  (37) Dqcu=g(t,u),  u(τ0)=u0≥0,
  exists for *t* ≥ *τ*_0_;
(iii) *g*(*t*, *u*) is nondecreasing in *t* for each *u*.

Then *V*(*t*_0_, *y*_0_ − *x*_0_) ≤ *u*_0_ implies

(38) V(t,y(t+η,τ0,y0)−x(t,t0,x0))≤r(t+η,τ0,u0)for t≥t0.

**Proof**

Define *m*(*t*) = *V*(*t*, *y*(*t* + *η*, *τ*_0_, *y*_0_) − *x*(*t*, *t*_0_, *x*_0_)) so that

(39) $$\begin{array}{c} m\left(t_{0}\right)=V\left(t_{0},y_{0}-x_{0}\right)\leq u_{0}. \end{array}$$

Let *z*(*t*, *t*_0_, *y*_0_ − *x*_0_) = *y*(*t* + *η*, *τ*_0_, *y*_0_) − *x*(*t*, *t*_0_, *x*_0_) so that

(40) Dqcz=f~(t,z)=[f(t+η,y(t+η))−f(t,x(t))],z(t0)=y0−x0,m(t)−m(t−h)hq =V(t,z(t))−V(t−h,z(t)−hq[f~(t,z)])hq  +V(t−h,z(t)−hq[f~(t,z)])−V(t−h,S(z,h,q))hq,

where $S\left(z,h,q\right)=z(t)-h^{q}(\tilde{f}(t,z))-\epsilon (h^{q})$. Since *V* is locally Lipschitzian in *x* and *L* > 0 is the Lipschitz constant and *ϵ*(*h* ^*q*^)/*h* ^*q*^ → 0 as *h* → 0, we have

(41) D+qcm(t)≤lim⁡h→0+⁡L||ϵ(hq)hq|| +limsup⁡h→0+⁡(V(t,y(t+η)−x(t))hq−V(t−h,y(t+η)−x(t))hq−hq[f(t+η,y(t+η))−f(t,x(t))]hq)≤g(t,m(t)).

By using Theorem 13 we obtain that

(42) m(t)=V(t,y(t+η,τ0,y0)−x(t,t0,x0))≤r(t+η,τ0,u0),for t≥t0.

### 4.2. Stability and Boundedness Criteria

A comparison principle is obtained by employing the notion of Lyapunov function together with the theory of differential inequalities. In this part, one can see Lyapunov-like function as a transformation which reduces the study of stability, boundedness, and Lagrange stability properties relative to ITD of a given complicated system to a relatively simpler scalar equation.

#### 4.2.1. Stability Criteria

**Theorem 15**

Assume that

(i) *V* ∈ *C*[ℝ_+_ × ℝ^*n*^, ℝ_+_], *V*(*t*, *x*) is locally Lipschitzian in *x* ∈ ℝ^*n*^, *g* ∈ *C*[ℝ_+_^2^, ℝ] and
  (43) D+qcV(t,y(t+η)−x(t))≤g(t,V(t,y(t+η)−x(t)))
(ii) the maximal solution *r*(*t*) = *r*(*t*, *τ*_0_, *u*_0_) of (37) exists for *t* ≥ *τ*_0_;
(iii) there exists *a*, *b* ∈ *𝒦* such that *b*(||*x*||) ≤ *V*(*t*, *x*) ≤ *a*(||*x*||) for (*t*, *x*) ∈ ℝ_+_ × *S*(*ρ*);
(iv) *g*(*t*, *u*) is nondecreasing in *t* for each *u* and *g*(*t*, 0) = 0;

Then the stability properties of the null solution of (37) imply the corresponding initial time difference stability properties of the solution *x*(*t*, *t*_0_, *x*_0_).

**Proof**

Assume that the null solution of (37) is equistable. Let 0 < *ϵ* < *ρ* be given. Then by definition of equistability given *b*(*ϵ*) > 0, *τ*_0_ ∈ ℝ_+_, there exist a *δ*_1_ = *δ*_1_(*ϵ*, *τ*_0_) such that

(44) $$\begin{array}{c} u\left(t\right)<b\left(\epsilon \right)\;\text{provided}\;\text{that}\;u_{0}<\delta_{1},\;t\geq \tau_{0}, \end{array}$$

where *u*(*t*, *τ*_0_, *u*_0_) is any solution of the (37). Choose *δ*_2_ = *δ*_2_(*ϵ*, *τ*_0_) > 0 as 0 < *a*(*δ*_2_) < *δ*_1_. Obviously lim⁡_(*τ*_0_,*y*_0_)→(*t*_0_,*x*_0_)_||*y*(*t* + *η*, *τ*_0_, *y*_0_) − *x*(*t*, *t*_0_, *x*_0_)|| = 0. Then given *ϵ* > 0 and *τ*_0_ ∈ ℝ_+_, $\tilde{\delta }=\tilde{\delta }(\epsilon ,\tau_{0})>0$ and *δ*_3_ = *δ*_3_(*ϵ*, *τ*_0_) > 0 such that

(45) ||y0−x0||<δ3,|η|<δ~  implies ||y(t+η)−x(t)||<ϵ for t0≤t≤τ0

Let *u*_0_ = *a*(||*y*_0_ − *x*_0_||) and choose *δ* = min⁡(*δ*_2_, *δ*_3_). Then we claim that

(46) $$\begin{array}{c} ||y\left(t+\eta \right)-x\left(t\right)||<\epsilon \;\text{provided}\;\text{that}||y_{0}-x_{0}||<\delta ,\\ \left|\eta \right|<\tilde{\delta }\;\text{for}\;\;t\geq t_{0}. \end{array}$$

If it is not true, from (45), there exist a *t*_1_ > *τ*_0_ and a solution *y*(*t*, *τ*_0_, *y*_0_) with ||*y*_0_ − *x*_0_|| < *δ* and $\left|\eta \right|<\tilde{\delta }$ such that

(47) $$\begin{array}{c} ||y\left(t_{1}+\eta \right)-x\left(t_{1}\right)||\geq \epsilon ,\\ ||y\left(t+\eta \right)-x\left(t\right)||<\epsilon ,\;\text{for}\;t_{0}\leq t<t_{1}. \end{array}$$

Moreover since ||*y*_0_ − *x*_0_|| < *δ*, by (iii) we have

(48) $$\begin{array}{c} V\left(t_{0},y_{0}-x_{0}\right)\leq a\left(||y_{0}-x_{0}||\right)<a\left(\delta \right)<\delta_{1}. \end{array}$$

Hence, by (i), (ii), (47) and, Theorem 14, we obtain the following estimate:

(49) $$\begin{array}{c} V\left(t,y\left(t+\eta \right)-x\left(t\right)\right)\leq r\left(t+\eta ,\tau_{0},u_{0}\right),\;t_{0}\leq t<t_{1}. \end{array}$$

Consequently, the relations (44), (47), (49), and (iii) lead to the contradiction

(50) b(ϵ)≤b(||y(t1+η)−x(t1)||)≤V(t1,y(t1+η)−x(t1))≤r(t1+η,τ0,u0)<b(ϵ).

which proves that the solution *x*(*t*, *t*_0_, *x*_0_) of (7) is equistable with ITD.

#### 4.2.2. Boundedness Criteria

In this section, Lagrange stability, which includes boundedness criteria, of fractional dynamic systems are discussed by employing comparison method.

**Theorem 16**

Let the assumption of Theorem 15 hold. Then the Lagrange stability properties of (37) imply the corresponding initial time difference Lagrange stability properties of (7).

**Proof**

We need to prove (B1) and (S3) for (7). Let *α* ≥ 0 be given, and let ||*y*_0_ − *x*_0_|| ≤ *α*. In view of (iii) *V*(*t*_0_, *y*_0_ − *x*_0_) ≤ *a*(*α*) = *α*_1_. Assume that (37) is Lagrange stable. It follows that (37) is bounded. Then, given *α*_1_ ≥ 0 and *τ*_0_ ∈ ℝ_+_ there exists a *β*_1_ = *β*_1_(*τ*_0_, *α*) such that

(51) $$\begin{array}{c} u_{0}\leq \alpha_{1}\;\;\text{imply}\;\;r\left(t,\tau_{0},u_{0}\right)<\beta_{1}. \end{array}$$

Moreover, *b*(*u*) → *∞* as *u* → *∞*; we can choose a *β* = *β*(*τ*_0_, *α*) verifying the relation

(52) $$\begin{array}{c} b\left(\beta \right)\geq \beta_{1}. \end{array}$$

Then from (45), given *β* > 0 and *τ*_0_ ∈ ℝ_+_, $\tilde{\delta }=\tilde{\delta }(\epsilon ,\tau_{0})>0$ and *δ*_1_ = *δ*_1_(*ϵ*, *τ*_0_) > 0 such that

(53) $$\begin{array}{c} ||y_{0}-x_{0}||<\delta_{1},\\ \left|\eta \right|<\tilde{\delta }\;\text{implies}||y\left(t+\eta \right)-x\left(t\right)||<\beta \;\text{for}\;\;t_{0}\leq t\leq \tau_{0}. \end{array}$$

Let *u*_0_ = *V*(*τ*_0_, *y*_0_ − *x*_0_). Then we claim that

(54) $$\begin{array}{c} ||y\left(t+\eta \right)-x\left(t\right)||<\beta \;\;\text{provided}\;\;\text{that}||y_{0}-x_{0}||<\alpha ,\\ \left|\eta \right|<\tilde{\delta }\;\text{for}\;\;t\geq t_{0}. \end{array}$$

If this were false, there would exist a t* > *τ*_0_ and a solution *y*(*t*, *τ*_0_, *y*_0_) of (7) such that $||\tilde{x}(t^{*}+\eta )-x(t^{*})||=\beta$. Consequently, the relations (49), (51), (52), and (iii) lead to the contradiction

(55) b(β)≤b(||y(t∗+η)−x(t∗)||)≤V(t∗,y(t∗+η)−x(t∗))≤r(t∗+η,τ0,u0)<β1≤b(β)

which proves that (B1) holds for (7).

To prove attractivity, we let *ϵ* > 0, *α* ≥ 0, *τ*_0_ ∈ ℝ_+_ be given and ||*y*_0_ − *x*_0_|| ≤ *α*. In view of (iii), *V*(*t*_0_, *y*_0_ − *x*_0_) ≤ *a*(*α*) = *α*_1_. Since (37) is attractive in the large with ITD, given *α*_1_ ≥ 0, *b*(*ϵ*) and *τ*_0_ ∈ ℝ_+_ there exist a *T* = *T*(*τ*_0_, *α*, *ϵ*) such that

(56) $$\begin{array}{c} u_{0}\leq \alpha_{1}\;\;\text{implies}\;\;u\left(t,\tau_{0},u_{0}\right)<b\left(\epsilon \right),\;\text{for}\;t\geq \tau_{0}+T. \end{array}$$

We have (49) from the proof of Theorem (45). Now, suppose that there exists a sequence {*t*_*k*_} ∈ ℝ_+_, *t*_*k*_ → *∞* as *k* → *∞*, *t*_*k*_ > *τ*_0_ + *T* and a solution *y*(*t*, *τ*_0_, *y*_0_) of (7) such that

(57) $$\begin{array}{c} ||y\left(t_{k}+\eta \right)-x\left(t_{k}\right)||\geq \epsilon . \end{array}$$

The relations (iii), (49), (56), and (57) lead to the contradiction,

(58) b(ϵ)≤b(||y(tk+η)−x(tk)||)≤V(tk,y(tk+η)−x(tk))≤r(tk+η,τ0,u0)<b(ϵ)

which proves that the system is attractive in the large with ITD.

## 5. Conclusion

Firstly, differential inequalities and a new comparison principle for fractional differential equations relative to initial time difference have been developed and then stability, boundedness criteria, and Lagrange stability relative to initial time difference have been proved by employing comparison method.

## References

[1] Lakshmikantham V, Leela S (1969). *Differential and Integral Inequalities*.

[2] Lakshmikantham V, Leela S, Martynyuk AA (1989). *, Stability Analysis of Nonlinear Systems*.

[3] Kilbas AA, Srivastava HM, Trujillo JJ (2006). *Theory and Applications of Fractional Differential Equations*.

[4] Podlubny I (1999). *Fractional Differential Equations*.

[5] Baleanu D, Diethelm K, Scalas E, Trujillo JJ (2012). *Fractional Calculus Models and Numerical Methods*.

[6] Lakshmikantham V, Leela S, Vasundhara Devi J (2009). *Theory of Fractional Dynamical Systems*.

[7] Li Y, Chen Y, Podlubny I (2010). Stability of fractional-order nonlinear dynamic systems: Lyapunov direct method and generalized Mittag-Leffler stability. *Computers and Mathematics with Applications*.

[8] Momani S, Hadid S (2004). Lyapunov stability solutions of fractional integrodifferential equations. *International Journal of Mathematics and Mathematical Sciences*.

[9] Qian D, Li C, Agarwal RP, Wong PJY (2010). Stability analysis of fractional differential system with Riemann-Liouville derivative. *Mathematical and Computer Modelling*.

[10] Shaw MD, Yakar C (1999). Generalized variation of parameters with initial time difference and a comparison
result in term Lyapunov-like functions. *International Journal of Non-Linear Differential Equations*.

[11] Yakar C, Cicek M, Bayram Gücen M (2012). Practical stability, boundedness criteria and Lagrange stability of fuzzy differential system. *Computers & Mathematics with Applications*.

[12] Song X, Li A, Wang Z (2010). Study on the stability of nonlinear differential equations with initial time difference. *Nonlinear Analysis: Real World Applications*.

[13] Li A, Lv W, Ye J (2011). Exponential and global stability of nonlinear dynamical systems relative to initial time difference. *Applied Mathematics and Computation*.

[14] Samko G, Kilbas AA, Marichev OI (1993). *Fractional Integrals and Derivatives: Theory and Applications*.

[15] Dirici Z, Mc Rae FA, Vasundhara Devi J (2012). On the existence and stability of solutions of Hybrid Caputo fractional differential equations. *Dynamics of Continuous, Discrete and Impulsive Systems*.

[16] Yakar C, Çiçek M (2011). Theory, methods and applications of initial time difference boundedness criteria and Lagrange stability in terms of two measures for nonlinear systems. *Hacettepe Journal of Mathematics and Statistics*.

